# A Case of T-Cell Large Granulocyte Lymphocytic Leukemia in Rheumatoid Arthritis

**DOI:** 10.7759/cureus.36266

**Published:** 2023-03-16

**Authors:** Ciji Robinson, Sruthi Ramanan, Harjinder Singh, Jasdeep S Minhas, Hazem Zebda

**Affiliations:** 1 Internal Medicine, Henry Ford Health System, Jackson, USA; 2 Medicine, St. George's University School of Medicine, St. George's, GRD; 3 Rheumatology, Henry Ford Health System, Detroit, USA

**Keywords:** t-cells, flow cytometry, lgl leukemia, leukemia, rheumatoid arthritis

## Abstract

T-cell large granulocyte lymphocytic (TLGL) leukemia is a lymphoproliferative disorder involving clonal expansion of cytotoxic T-cells and subsequent cytopenia, most notably neutropenia, as well as splenomegaly. TLGL leukemia is commonly associated with autoimmune disorders, most commonly rheumatoid arthritis (RA). We present a case of a 54-year-old female with a past medical history of seropositive RA who was lost to follow-up and thus was not on any active treatment for RA for several years. She returned to the clinic with worsening joint pain, swelling, and stiffness involving multiple joints. Screen laboratory work revealed an absolute neutrophil count (ANC) of 0.19 K/uL, indicating severe neutropenia. This finding prompted further workup, for which our patient was ultimately diagnosed with TLGL leukemia. Proper treatment and control of inflammation in RA are important not only to preserve joint function and vitality but also to prevent rare sequela of untreated autoimmune disorders, as was the case in our patient.

## Introduction

T-cell large granulocyte lymphocytic (TLGL) leukemia is a lymphoproliferative disorder that can occur in association with rheumatoid arthritis (RA) [1]. The disease course is typically indolent and thus can develop over a long time course prior to symptoms becoming apparent [2]. It is an extremely rare disease with an incidence of 0.2 per 1,000,000 in the United States [3]. Routine laboratory work including a complete blood count with a differential indicating neutropenia is vital in raising initial clinical suspicion for the disease. We present a case of TLGL leukemia in an RA patient to add to the existing literature given the rarity of the disease.

## Case presentation

**Figure 1:**
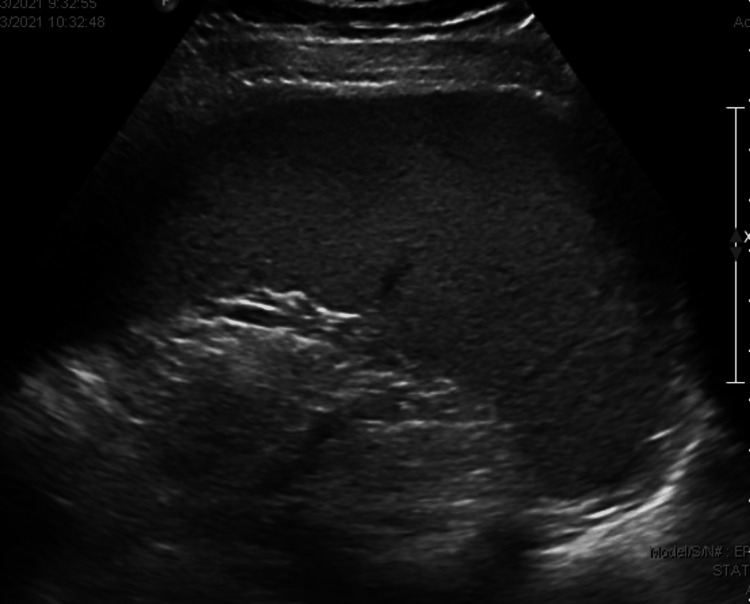
Abdominal ultrasound revealing mild splenomegaly

**Figure 2:**
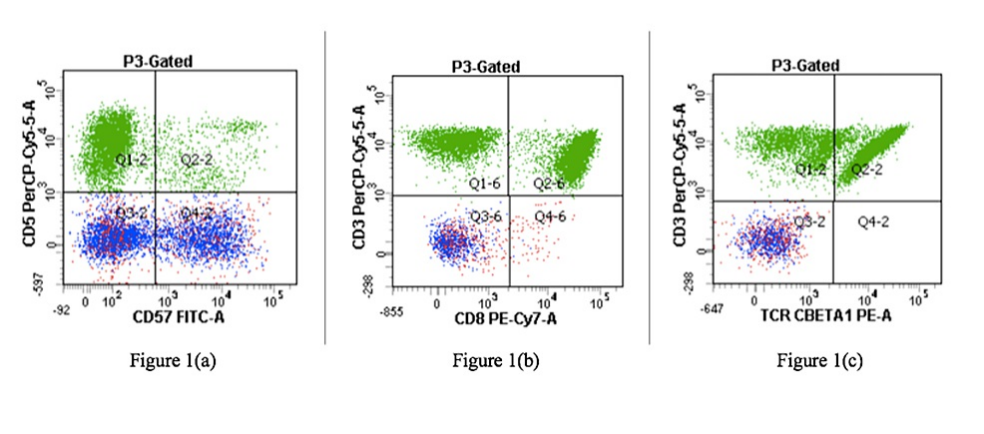
(a-c) Our patient’s flow cytometry with remarkable TCR gene rearrangement and CD3(+) CD8(+) CD57(+) phenotype TCR: T-cell receptor

We present a case of a 54-year-old female with a past medical history of seropositive RA presenting to the rheumatology clinic for increased pain, swelling, and stiffness involving numerous joints. She was first evaluated at the rheumatology clinic three years prior to presentation and was diagnosed with seropositive rheumatoid arthritis at that time. She was prescribed methotrexate but was lost to follow-up and did not return or begin the medication. Upon our patient’s return to the clinic, she was found to have synovitis involving the proximal interphalangeal phalange (PIP) joints and metacarpophalangeal (MCP) joints bilaterally, as well as bilateral elbows, wrists, and knees with reduced range of motion. She was then prescribed methotrexate to begin again and to have a close follow-up in the clinic. At that time, routine laboratory work was also obtained for medication monitoring purposes. Our patient was found to have an absolute neutrophil count (ANC) of 0.19 K/uL, indicating severe neutropenia (Table 1). She was subsequently started on a long course of steroids with an eventual taper; she also underwent an abdominal ultrasound that was remarkable for mild splenomegaly (Figure 1). Our patient underwent bone marrow biopsy and subsequent flow cytometry, which ultimately lead to the diagnosis of T-cell large granular lymphocytic (TLGL) leukemia given the CD3(+) CD8(+) CD57(+) phenotype with positive T-cell receptor (TCR) gene rearrangement (Figure 2). Given that treatment of TLGL leukemia is to treat the underlying autoimmune disorder, our patient was continued on methotrexate and was to continue treatment with oral prednisone. Her condition showed improvement with an increase of her ANC > 0.5 K/uL, which continues to be closely monitored.

**Table 1:** Our patient’s complete blood count with differential WBC: white blood cell, RBC: red blood cell, MCV: mean corpuscular volume, MCH: mean corpuscular hemoglobin, MCHC: mean corpuscular hemoglobin concentration, RDW: red blood cell distribution width

Component	Reference range	Result
WBC count	3.8-10.6 K/uL	1.9 K/uL
RBC count	4.15-5.55 M/uL	4.29 M/uL
Hemoglobin	12.0-15.0 g/dL	13.2 g/dL
Hematocrit	36%-46%	39%
MCV	80-100 fl	90.9 fl
MCH	26-34 pg	30.8 pg
MCHC	31-37 g/dL	33.8 g/dL
RDW	<14.5%	12.9%
Platelet count	150-450 K/uL	142 K/uL
Neutrophil %	%	10%
Band %	%	6%
Lymphocyte %	%	78%
Monocyte %	%	4%
Eosinophil %	%	0%
Basophil %	%	2%
Neutrophil, absolute	1.80-7.70 K/uL	0.19 K/uL
Band, absolute	0.00-0.80 K/uL	0.11 K/uL
Lymphocytes, absolute	1.10-4.00 K/uL	1.48 K/uL
Monocytes, absolute	0.00-0.80 K/uL	0.08 K/uL
Eosinophils, absolute	0.00-0.70 K/uL	0 K/uL
Basophils, absolute	0.00-0.20 K/uL	0.04 K/uL

## Discussion

TLGL leukemia is an extremely rare lymphoproliferative disorder that can occur in association with autoimmune and hematologic disorders, most commonly RA [1]. The pathophysiology behind TLGL leukemia is poorly understood but has been noted to be associated with dysregulated signaling related to the mass proliferation of immune cells and dysregulated apoptotic pathways associated with chronic inflammation [4]. First-line treatments include immunosuppressive therapy including methotrexate, cyclophosphamide, and cyclosporin A to treat the underlying inflammatory process [4]. If refractory to the aforementioned treatments, certain chemotherapeutic agents or other immunomodulating agents may be used [4].

Once neutropenia is identified in patients with RA, further investigation is warranted. A peripheral blood smear is a safe and noninvasive next step that can identify TLGL cells and quantify them to help guide the diagnosis [5]. The presence of more than 0.25 × 10^9^/L of LGL establishes the diagnosis of LGL leukemia; however, most patients exceed this number and range anywhere between 2 and 10 × 10^9^/L [5]. Bone marrow aspirate can then be obtained, followed by flow cytometry, to establish the TLGL cell phenotype, which can also guide the diagnosis [6,7]. The phenotype of CD3(+), CD8(+), CD57(+), CD56(−), CD28(−), and TCR-αβ(+) is seen in 80%-90% of TLGL leukemia cells [8]. Abdominal ultrasound revealing splenomegaly can also aid in supporting the diagnosis [1].

Our patient’s presentation with progressed RA, critically low neutrophil count, mild splenomegaly, and the CD3+ CD8(+) CD57(+) phenotype with positive T-cell receptor (TCR) gene rearrangement of TLGL cells all help support the diagnosis of TLGL leukemia.

## Conclusions

TLGL leukemia is a rare sequela of progressed RA, and the identification of neutropenia in RA patients warrants further investigation as this could be the first sign of the disease. Given the rarity of TLGL leukemia, increasing awareness by case report publishing describing workup and treatment/progression of patients is important to add to the existing literature, which was the aim of this report.
